# Population Pharmacokinetic, Pharmacogenetic, and Pharmacodynamic Analysis of Cyclophosphamide in Ethiopian Breast Cancer Patients

**DOI:** 10.3389/fphar.2020.00406

**Published:** 2020-04-23

**Authors:** Jemal Hussien Ahmed, Eyasu Makonnen, Ronald Kuteesa Bisaso, Jackson Kijumba Mukonzo, Alan Fotoohi, Abraham Aseffa, Rawleigh Howe, Moustapha Hassan, Eleni Aklillu

**Affiliations:** ^1^ Department of Pharmacology and Clinical Pharmacy, Addis Ababa University, Addis Ababa, Ethiopia; ^2^ Division of Clinical Pharmacology, Department of Laboratory Medicine, Karolinska Institutet, Karolinska University Hospital, Stockholm, Sweden; ^3^ Center for Innovative Drug Development and Therapeutic Trials, Addis Ababa University, Addis Ababa, Ethiopia; ^4^ Department of Pharmacology and Therapeutics, College of Health Sciences, Makerere University, Kampala, Uganda; ^5^ Division of Clinical Pharmacology, Department of Medicine, Karolinska Institutet, Stockholm, Sweden; ^6^ Non-Communicable Diseases (NCD) Research Directorate, Armauer Hansen Research Institute, Addis Ababa, Ethiopia; ^7^ Experimental Cancer Medicine (ECM), Clinical Research Center (KFC), Department of Laboratory Medicine, Karolinska Institutet, Stockholm, Sweden

**Keywords:** cyclophosphamide, pharmacokinetics, CYP3A5, CYP2C9, body surface area, pharmacodynamics, breast cancer

## Abstract

Cyclophosphamide (CPA) containing chemotherapy regimen is the standard of care for breast cancer treatment in sub-Saharan Africa. Wide inter-individual variations in pharmacokinetics (PK) of cyclophosphamide (CPA) influence the efficacy and toxicity of CPA containing chemotherapy. Data on the pharmacokinetics (PK) profile of CPA and its covariates among black African patients is lacking. We investigated population pharmacokinetic/pharmacogenetic/pharmacodynamic (PK-PG-PD) of CPA in Ethiopian breast cancer patients. During the first cycle of CPA-based chemotherapy, the population PK parameters for CPA were determined in 267 breast cancer patients. Absolute neutrophil count was recorded at baseline and day 20 post-CPA administration. A population PK and covariate model analysis was performed using non-linear mixed effects modeling. Semi-mechanistic and empiric drug response models were explored to describe the relationship between the area under concentration-time curve (AUC), and neutrophil toxicity. One compartment model better described CPA PK with population clearance and apparent volume of distribution (V_D_) of 5.41 L/h and 46.5 L, respectively. Inter-patient variability in CPA clearance was 54.5%. Patients carrying CYP3A5*3 or *6 alleles had lower elimination rate constant and longer half-life compared to wild type carriers. CYP2C9 *2 or *3 carriers were associated with increased clearance of CPA. Patients who received 500 mg/m^2^ based CPA regimen were associated with a 32.3% lower than average clearance and 37.1% lower than average V_D_ compared to patients who received 600 mg/m^2^. A 0.1 m^2^ unit increase in body surface area (BSA) was associated with a 5.6% increment in V_D_. The mean V_D_ (33.5 L) in underweight group (BMI < 18.5 kg/m^2^) was significantly lower compared to those of overweight (48.1 L) or obese patients (51.9 L) (*p* < 0.001). AUC of CPA was positively correlated with neutropenic toxicity. In conclusion, we report large between-patient variability in clearance of CPA*. CYP3A5* and *CYP2C9* genotypes, BSA, BMI, and CPA dosage regimen influence PK of CPA. Plasma CPA exposure positively predicts chemotherapy-associated neutropenic toxicity.

## Introduction

Cyclophosphamide (CPA) is a cornerstone of combination chemotherapy for the treatment of breast cancer (Stearns et al., 2004). Wide inter-individual variations in the pharmacokinetics (PK) of CPA and its treatment outcome including safety have been reported (de Jonge et al., 2005). CPA is a prodrug and requires bio-activation in the liver to 4-hydroxy-cyclophosphamide (4-OH-CPA), which is subsequently converted to the ultimate alkylating metabolite, phosphoramide mustard, and acrolein, a urotoxic metabolite (Ekhart et al., 2008). Various cytochrome P450 (CYP) enzymes have been implicated to catalyze the 4-hydroxylation of CPA to 4-OH-CPA including *CYP2B6* (Xie et al., 2003), *CYP2C9*, and *CYP3A4/5* (Roy et al., 1999), *CYP2C19* (Griskevicius et al., 2003; Timm et al., 2005), and *CYP2J2* (El-Serafi et al., 2015a). These enzymes are genetically polymorphic and may contribute to interindividual variation in CPA metabolic disposition and clinical response including chemotherapy-induced toxicities (Nakajima et al., 2007; Zanger et al., 2007; Ekhart et al., 2008). Apart from genetic factors, other patient-specific characteristics such as disease status, body weight, age, body surface area, and hepatic and renal function status may influence CPA plasma exposure (de Jonge et al., 2005). A decrease in CPA clearance with increased body weight (Powis et al., 1987), impaired hepatic (de Jonge et al., 2005), or renal function (Haubitz et al., 2002) resulting in an increased systemic drug exposure is reported previously.

Identifying factors influencing PK parameters and exposure-toxicity relationship is critical for CPA dose optimization and personalized chemotherapy. The population pharmacokinetics of high dose CPA (4,000–6,000 mg/m^2^), has previously been described by a time-dependent or concentration-dependent PK models (Chen et al., 1995; Chen et al., 1997). CPA auto-induction has been modeled by a mechanistic enzyme turnover model based on the data of both CPA and 4-hydroxycyclophosphamide (Hassan et al., 1999). On the other hand, Huitema *et al*. reported a PK model for the bioactivation route of CPA incorporating both auto-induction and drug interaction between CPA and Thiotepa (Huitema et al., 2001).

CPA-based chemotherapy suppresses the hematopoietic system, impairing host protective mechanisms and limiting the doses of chemotherapy that can be tolerated. Neutropenia remains the major adverse event of CPA at conventional doses and the main concern in the delivery of CPA-based chemotherapy (Crawford et al., 2004). CPA-based chemotherapy-associated neutropenia is the primary reason for change in the schedule of chemotherapy delivery to cancer patients (Kozma et al., 2012). Particularly, patients manifesting febrile neutropenia, a serious complication of chemotherapy with risk of confusion, cardiac complications, hypotension, respiratory and renal failure, and even death, often require hospitalization and administration of antibiotics (Weycker et al., 2014). Chemotherapy-induced hematological adverse events cause a substantial economic burden on patients, caregivers, and society at large (Liou et al., 2012). Consequently, one aspect of optimizing cancer chemotherapy involves describing neutropenic toxicity as a function of drug exposure or its pharmacokinetic parameters.

Ethnic differences in anticancer drug disposition is an important factor accounting for population variation in treatment response and tolerability (O’Donnell and Dolan, 2009). Hence PK, pharmacogenetics (PG) and exposure-toxicity relationship of CPA in various populations require definition. CPA has remained a stable component in many of the chemotherapy combinations used in breast cancer CPA in sub-Saharan Africa including Ethiopia. To our knowledge, the PK profile of CPA and its covariates among black African breast cancer patients is yet to be investigated. Furthermore, the exposure-myelosuppressive toxicity relationship of CPA in black African population is not studied. Therefore, this study was aimed to investigate the population pharmacokinetics and pharmacodynamics (PD) of CPA and identify potential covariates including pharmacogenetic markers influencing PK of CPA in Ethiopian breast cancer patients.

## Materials and Methods

### Patients

A total of 267 female breast cancer patients were enrolled from the radiotherapy center of Tikur Anbessa specialized hospital, Addis Ababa, Ethiopia. The study protocol was approved by Institutional Review Board of the College of Health Sciences, Addis Ababa University (Ref No: 011/16/2016), Armauer Hansen Research Institute Ethical Review Committee (Ref No: PO26/16), and National Research Ethics Review Committee of the Federal Democratic Republic of Ethiopia (Ref No: 3.10/235/2017). Written informed consent was obtained from each patient before participation in the study. Breast cancer patients receiving CPA containing chemotherapy regimen with conventional dosage (500–600 mg/m^2^) were included. The common CPA-based chemotherapy regimens in the outpatient care ward of the radiotherapy center were AC-T (Adriamycin 60 mg/m^2^ and cyclophosphamide 600 mg/m^2^ followed by Taxol 175 mg/m^2^), FAC [5-fluorouracil 500 mg/m^2^, Adriamycin (doxorubicin) 50 mg/m^2^, and cyclophosphamide 500 mg/m^2^] and AC (Adriamycin 50 mg/m^2^ and cyclophosphamide 600 mg/m^2^). Patients were stratified into 600 mg/m^2^ CPA based regimen (AC and AC-T) and 500 mg/m^2^ CPA based regimen (FAC). The individual dose of each drug was calculated based on body surface area (BSA) of the patient and CPA was infused i.v over 30 min.

The criteria for inclusion were white blood cells count (WBC) ≥ 3,000/mm^3^; absolute neutrophil count (ANC) ≥ 1,500/mm^3^; and platelet count (PLT) ≥ 100,000/mm^3^; aspartate aminotransferase (AST), alanine aminotransferase (ALT), and serum creatinine (SCr) ≤ 2.5 times the upper limits of normal; Karnofsky’s performance score of at least 70% at presentation. For all included patients, socio-demographic data (age, weight, height, etc) and clinical profiles (stage of breast cancer, nodal status, tumor size, degree of differentiation, co-morbidity status, co-medication, pretreatment liver function [AST, ALT, alkaline phosphatase (ALP)], and renal function estimates [SCr, blood urea nitrogen (BUN)] were collected from patient medical charts. All patients were also given prophylactic pre-medications that comprised cimetidine (400 mg i.v), dexamethasone (8 mg i.v), and ondansetron (8 mg i.v) prior to infusion of chemotherapy.

### Blood Sampling

During the first cycle of CPA-containing chemotherapy, blood samples were drawn in ethylenediaminetetraacetic acid (EDTA) tubes for genotyping and heparin tubes for cyclophosphamide (CPA) PK analysis. From all 267 patients, two blood samples were collected before CPA infusion starts (0 h) for genotyping (2 ml) and CPA PK (2 ml). From 250 patients, additional blood samples (2 ml) were collected at 1(2), 3(4), and/or 5(6) h after CPA infusion starts. From the remaining 17 patients, additional PK blood samples were also collected at 4, 8, 12, and 22 h post-CPA infusion initiation. These patients were admitted to receive their first cycle chemotherapy and monitored overnight. All PK blood samples were centrifuged within 30 min of collection at 3,500xg and 4°C for 3 min to separate the plasma (Nakajima et al., 2007). Both PK plasma and genotyping whole blood samples were then stored at −80°C until analysis.

### Determination of Plasma Cyclophosphamide Concentrations

The plasma concentrations of CPA was determined as described previously with some modifications (El-Serafi et al., 2015b). To each 250 µl of thawed plasma samples, 25 ml of internal standard (ifosfamide, 20 mM, Vnr079111, Denmark), and 1.25 ml ethyl acetate were added. The mixture was briefly vortexed for 15 s and then centrifuged at 5,000 rpm, 4°C for 10 min. The organic fraction was transferred into a clean tube and evaporated to dryness under vacuum centrifuge. The residue was then dissolved in 100 µl of mobile phase, and the resulting solution was put in ultrasonic bath for 15 s. Fifty micro-liter of the solution was then injected into high performance liquid chromatography (HPLC) system. The HPLC system consisted of LKB-2150 Pump, Gilson-234 Auto-Injector with a 100 μl sample loop, ZORBAX Extend-C18 column (150 × 4.6 mm, 3.5 μm, Agilent, Santa Clara, CA, USA) with a C18 guard column (Agilent, Santa Clara, CA, USA) and Milton Roy UV Spectro-Monitor 3100 detector (Pont-Saint-Pierre, France). The mobile phase consisted of acetonitrile/0.05 M KH_2_PO_4_ buffer (24:76 v/v, pH 2.8). The flow rate was 1 ml/min and detection was monitored at 195 nm. Data acquisition and processing were done using Clarity Chromatographic software (version 6.0, DataApex, Prague, Czech Republic). Under these conditions, the retention time was 5.5 and 6 min for ifosfamide and CPA, respectively. The standard curve was constructed using CPA free plasma spiked with increasing concentrations (1–2,000 µmol/L) of standard CPA (Sigma Aldrich Co, China). The quantification limit of CPA was 5 µmol/L. The standard curve was linear in the range of 5–1,000 µmol/L (r = 0.998). Quality control samples (three samples, at a concentration of 25, 100, and 500 µmol/L) were included routinely at each run.

### Genotyping

Genomic DNA was isolated from peripheral leukocytes in whole blood samples using QIAamp DNA Midi Kit (Qiagen GmbH, Hilden, Germany) following the manufacturer’s instruction. Genotyping was performed using TaqMan^®^ drug metabolism genotyping assay reagents (Applied Biosystems Genotyping Assays) for allelic discrimination as described previously (Ahmed et al., 2019), with the following ID numbers for each SNP: C_7817765_60 for CYP2B6*6 (rs3745274), C_26201809_30 for CYP3A5*3 (rs776746), C_30203950_10 for CYP3A5*6 (rs10264272), C_25625805_10 for CYP2C9*2 (rs1799853), C_27104892_10 for CYP2C9*3 (rs1057910), C_25986767_70 for CYP2C19*2 (rs4244285), C_27861809_10 for CYP2C19*3 (rs4986893), C_9581699_80 for CYP2J2*7 (rs890293), C_8890131_30 for POR*28 (rs1057868), and C_11711730_20 for *ABCB1* (rs3842). The genomic DNA samples were amplified in 96-well plates on QuantStudio™ 12K Flex Real-Time PCR system (Applied Biosystems Life Technologies Holding, Singapore). The final volume for each reaction was 10 μl, consisting of TaqMan^®^ fast advanced master mix (Applied Biosystems, Waltham, MA, USA), TaqMan 20X/40X drug metabolism genotyping assays mix (Applied Biosystems, USA), and genomic DNA. The PCR conditions consisted of an initial step at 60°C for 30 s, hold stage at 95°C for 10 min and PCR stage for 40 cycles, step 1 with 95°C for 15 and step 2 with 60°C for 1 min and after read stage with 60°C for 30 s. The characterized SNPs were selected on the basis of their potential to influence the functionality of enzymes to affect the disposition of CPA.

### Population Pharmacokinetic Modeling

A population PK model of CPA was built using nonlinear mixed-effect modeling (NONMEM) program (version 7.30, ICON development solutions, Gaithersburg, Maryland). Additional software tools were also used as a workbench to facilitate the use of NONMEM including PsN (Lindbom et al., 2005) (version 3.4.2), Xpose (R Core Team, 2018) (version 4.5.0), and Pirana (Keizer et al., 2011) (version 2.9.6). A one, two, and three-compartment models were fitted to the data set in that order. Differential equations were used to specify the compartment in NONMEM using ADVAN6 subroutine. First-order conditional estimation with interaction (FOCE-I) was used to estimate model parameters. The structural model was parameterized with clearance (CL), inter-compartmental clearance (Q), and compartmental volume of distribution (V_n_), where n is the number of compartments. Inter-individual variability (IIV) in model parameters was assumed to be log-normally distributed with mean zero and variance ω^2^ (OMEGA squared) and specified by exponential functions [Exp(ω^2^)-1]. Additive, proportional and combined proportional and additive residual error models were explored to account for within-subject variability, experimental errors, and model misspecification. The model with the lowest objective function value (OFV) was presumed to fit the data better. Residual-based and prediction-based goodness of fit plots (GOF) were also visually examined to determine the best fitting base model.

Based on the base model, covariate analysis was performed using stepwise covariate modeling (SCM) in NONMEM with the help of PsN SCM configuration file. The variables selected in covariate analysis were socio-demographics (BSA and BMI), CPA dosage regimen (500 *vs.* 600 mg/m^2^ based CPA), genotype (*CYP2BC6, CYP2C9, CYP2C19, CYP3A5, CYP2J2*, *POR*, and *ABCB1*), comorbidity status (presence of cardiovascular diseases or HIV), and baseline organ function estimates (AST, ALT, ALP, SCr, BUN). The variables were selected based on clinical relevance or suggested impact on CPA disposition.

In the first phase of the SCM, covariates were added to the base model in a stepwise manner based on statistical significance (decrease in OFV). The effect of each potential covariate on each parameter was independently tested with a 5% significance level corresponding to a drop in OFV > 3.84 for 1° of freedom. The covariate with the largest change in OFV is added to the model. The procedure was repeated for all covariates until no more significant covariates are identified. In the backward elimination step, the level of significance was set at 1% threshold corresponding to a change in OFV > 6.63 for 1° of freedom or 9.210 for 2° of freedom. The covariates selected in the forward step are removed from the model one at a time. The covariates which caused the largest statistically significant increase in OFV upon removal were retained in the model. Continuous covariates such as BSA and BMI were centered on their median values and the estimated shape parameter, THETA1, which explains the relationship between population PK parameters and covariates according to the equation used in the model, was coded as exponential factor as designated in equation 1:

(1) $${\text{Clearance (CL)}=\text{CL}}_{\text{pop}}\times (\text{BSA/median}{)}^{\text{ THETA}1},$$

where, CL_pop_ represents the population clearance value for patients with median BSA and THETA1 is the exponential factor for BSA, which describes the relationship with CL. Binary covariates were coded as 0 (wild type/no) and 1 (variant/yes) and modeled as designated in equation 2, where the shape parameter, THETA2, represents the proportional change in clearance compared to the reference category (*e.g*., wild-type genotype).

(2) $$\text{CL}={\text{CL}}_{\text{pop}}\times (1+\text{THETA}2*\text{genotype}),$$

Interpatient variability (IIV) in plasma CPA PK parameters were described by percentage coefficient of variation (CV) given by %CV = SQRT(Exp(ω^2^)-1). The predictive performance of the final covariate model was evaluated using prediction corrected visual predictive check (pcVPC). At least 200 simulated datasets were created using the final covariate model. Median, the 5^th^ and 95^th^ percentile of the observed data were compared to the 95% prediction intervals for the simulated data to ascertain the predictive performance of the final covariate model.

### Modeling of Neutropenic Toxicity

Absolute neutrophil count (ANC) at baseline and at day 20 post-CPA administration (in the first cycle) were used to develop the neutropenic toxicity model. A semi-mechanistic (indirect response) and empiric (direct drug effect) PD models (Felmlee et al., 2012; Woo et al., 2017) were applied to describe CPA-related neutropenic toxicity. Initially, the indirect response model was explored. It was assumed that cells from myeloblasts to myelocytes are chemotherapy-sensitive and bone marrow leukocyte inhibition depends on sensitive cell exposure (unlike the stem cells which divide but are less sensitive to chemotherapy than mitotic hematopoietic precursors). Thus, we chose the inhibition of cell production model (Woo et al., 2017) as described by the following schematic representation and equation (Equation 3), where k_in_ is the zero-order production rate constant, *i.e*., the rate of entry of neutrophils into the circulating pool, k_out_ (K_in_/ANC_o_) is the first-order elimination rate constant, *i.e*., the rate of disappearance of neutrophils from the circulating pool. E_max_ is defined as the maximum fractional factor of inhibition or a factor that inhibits neutrophil synthesis as an anticancer chemotherapy effect (0 < E_max_ ≤ 1). ANC is the drug effect (neutrophil count at any time t) and ANC_o_ is the baseline neutrophil count. Since area under the concentration time curve (AUC) represents the systemic exposure, we incorporated the AUC, from time zero to day 20 after the first dose of CPA in the PD model to describe the relationship between AUC and drug effect (Equation 3). AUC_50_ represents the area under the curve associated with 50% of the maximum effect

→Kin(in the circulating pool)  Neutrophils→Kout

(3) $$\frac{d(ANC)}{dt}=K_{in}\left(1-\frac{E_{\max}\times AUC}{AUC_{50}+AUC}\right)-K_{out}\times ANC,$$

For AUC significantly less than AUC_50_ (*i.e*., AUC_50_ ≫ AUC), AUC in the denominator of equation 3 can be considered negligible and the relationship can be reduced to Equation 4, with *m* = E_max_/AUC_50_ is the slope of the relationship. Subsequently, K_in_, slope (*m*) of the relationship and ANC_o_ were estimated using NONMEM.

(4) $$\frac{d(ANC)}{dt}={\text{K}}_{\text{in}}(1-\text{m}\times \text{AUC})-{\text{K}}_{\text{out}}\times \text{ANC}$$

On the other hand, empiric model (linear direct drug response model) (Felmlee et al., 2012) was also separately adapted to the AUC data to correlate neutrophil count according to Equation 5, from which ANC_o_ and slope (*m*) were estimated using NONMEM.

(5) $$\text{Y}={\text{ANC}}_{\text{o}}-\text{slope }(m)\times \text{AUC}$$

where, Y represents the drug effect (*i.e*., absolute neutrophil count at any time t).

### Statistical Analysis

χ^2^-test was used to evaluate the genetic structure of the patient population (Hardy–Weinberg equilibrium). Descriptive statistics were computed to explore the demographic characteristics, clinical profiles, genotype frequencies of participants. Empirical Bayesian estimates of PK parameters were described as mean ± standard deviations (SD) or median with interquartile range (IQR). Inter-patient variability (IIV) in CPA PK parameters was described by coefficient of variation % CV = SQRT(Exp(ω^2^)-1), where ω^2^ is OMEGA squared representing variance in the PK parameters. One-way ANOVA or independent sample t-test was also used in SPSS for Windows (version 21.0), to test for association between derived PK parameters (AUC, K_el_, and t_1/2_) and genotype or other patient-specific factors (*i.e.,* BMI, BSA, etc). Multiple comparisons were performed based on Tukey tests. Graphs were prepared using R software (version 3.5.1) (R Core Team, 2018).

## Results

### Socio-Demographic and Clinical Profiles

CPA concentration was determined from a total of 532 plasma samples obtained from 267 female patients with breast cancer (average 2 plasma samples per patient). The socio-demographic and baseline laboratory results are summarized in **Table 1**. The median dose of CPA given to patients per cycle was 777.5 (range 600–1,000 mg) and 930 mg (Range 650–1,150 mg), respectively for 500 mg/m^2^ and 600 mg/m^2^ based regimen group [the overall median CPA dose given per cycle was 870 mg (range 600–1,150 mg)]. The genotype and allelic frequency distributions of candidate CPA metabolizing enzymes’ and transporter genes are presented in **Table 2**. All the genotype frequencies were in line with Hardy–Weinberg equilibrium (*p* > 0.05).

**Table 1:** Baseline socio-demographic, clinical and laboratory values of participants.

Variables^*^	Values (mean/median)	Values N (%)
Socio-demographic		
Age (years; median + IQR)	38 (33 – 48)	–
BSA (m^2^; mean ± SD*)*	1.59 ± 0.20	–
BMI (kg/m^2^; mean ± SD)	23.78 ± 4.8	–
Baseline differential blood parameters		
Neutrophils (10^3^ cells/mm^3^; median + IQR)	3645.5 (2645 - 4765)	–
Hemoglobin (g/dL; mean + SD)	13.91 ± 1.30	–
Platelet (10^3^ cells/mm^3^; median + IQR)	294 (249 – 346)	–
Baseline Liver function estimates		
ALT (U/L; median (IQR)	18 (13 - 27)	–
AST (U/L; median (IQR)	24 (19-31)	–
ALP (U/L; median (IQR)	208.5 (155 - 293)	–
Baseline Renal function estimates		
SCr (mg/dL; mean (± SD)	0.91 ± 0.2	–
BUN (mg/dL; median (IQR)	18.80 (14 – 24)	–
CRCL (mL/min; median (IQR)	76.40 (60.5 - 93.4)	–
CPA regimen		
500 mg/m^2^ based	–	106 (39.7)
600 mg/m^2^ based	–	161 (60.3)
Cardiovascular co morbidity		
Yes	–	23 (8.6)
No	–	244 (91.4)
Presence of HIV		
Yes	–	9 (3.4)
No	–	258 (96.6)

*ALP, Alkaline phosphatase; ALT, alanine aminotransferase; ANC, absolute neutrophil count; AST, aspartate aminotransferase; BUN, Blood urea nitrogen; BSA, body surface area; CRCL, Creatinine clearance; Hgb, hemoglobin; HCT, hematocrit; IQR, inter quartile range; PLT, platelet coun; SCr, Serum creatinine; SD, standard deviation; WBC, white blood cell count.

**Table 2:** Genotype and allele frequency distribution of candidate genes relevant to CPA activation or transport.

Gene	Genotype	Genotype frequency	Minor Allele frequency	
		N (%)	Allele	N (%)
*CYP2B6*	*1/*1	114 (42.7)		
	*1/*6	121 (45.3)	*6	185 (34.6)
	*6/*6	32 (12)		
*CYP2C9*	*1/*1	229 (85.8)		
	*1/*2 or *1/*3	35 (13.1)	*2	36 (6.74)
	*2/*2 or *3/*3	3 (1.1)	*3	6 (1.12)
*CYP2C19*	*1/*1	188 (70.4)		
	*1/*2 or *1/*3	74 (27.7)	*2	80 (15.0)
	*2/*2 or *3/*3	5 (1.9)	*3	8 (1.5)
*CYP3A5*	*1/*1	32 (12)		
	*1/*3 or *1/*6	105 (39.3)	*3	325 (60.9)
	*3/*3 or *6/*6	130 (48.7)	*6	75 (14.0)
*CYP2J2*	*1/*1		197 (73.8)	
	*1/*7	62 (23.2)	*7	78 (14.6)
	*7/*7	8 (3.0)		
*POR*	*1/*1	189 (70.8)		
	*1/*28	69 (25.8)	*28	87 (16.3)
	*28/*28	9 (3.4)		
*ABCB1* (rs3842, A>G)	*AA*	193 (79.1)		
	*AG*	44 (18)	*G*	58 (11.9)
	*GG*	7 (2.9)		

A star (*) followed by a unique Arabic numeral is a standard Allele nomenclature system in pharmacogenetics for specific alleles and used to denote nucleotide changes in a reference gene sequence. Wild type or normal gene sequence is usually denoted by *1/*1.

### Population Pharmacokinetic Modeling

CPA plasma concentration data were better described by one-compartment PK model. Additive error model of residual variability performed better than any of the other error models, *i.e*., proportional or exponential or combined error models. The parameter estimates of the population PK model of CPA are shown in **Table 3**. In the final model, the typical values for clearance and apparent volume of distribution of CPA were 5.41 L/h and 46.5 L, respectively. The estimated inter-individual variability (IIV) in clearance was 54.5% (38.9% and 49.3% for 500 and 600 mg/m^2^ group, respectively) (**Table 3**). The mean clearance values of CPA were 3.97 and 5.81 L/h for patients who received 500 and 600 mg/m^2^ based CPA regimen, respectively.

**Table 3:** Population pharmacokinetic parameter estimates.

Description	OFV^δ^	Population estimate		Between subject variability (%SE)
		Parameter	%SE^γ^	
**Base model**	2372.44	CL	5.34 (4.4)	54.5% (8)
		V_D_	30.4 (8.4)	44.7% (21)
**Final model**	2257.13	CL	5.41 (8.4)	46.4% (10)
		V_D_	46.5 (13.2)	35.9% (11)
**Residual variability**				
Additive error 1	1.54 mg/L (24%)			
Additive error 2	0.0001 mg/mL			
**Derived PK Parameters**				
Elimination constant (K_el_, hr^-1^; median, IQR^†^)	0.126 (0.1- 0.166)			
Half-life (hr, median, IQR)	5.48 (4.18 – 6.91)			
AUC^‡^_0-day20_ (µmol.h/L; median, IQR)	565.7 (444.9 - 828.1)			

^‡^AUC area under the curve; ^†^IQR inter-quartile range; **^δ^**OFV objective function value; ^γ^SE standard error.

**Figure 1:**
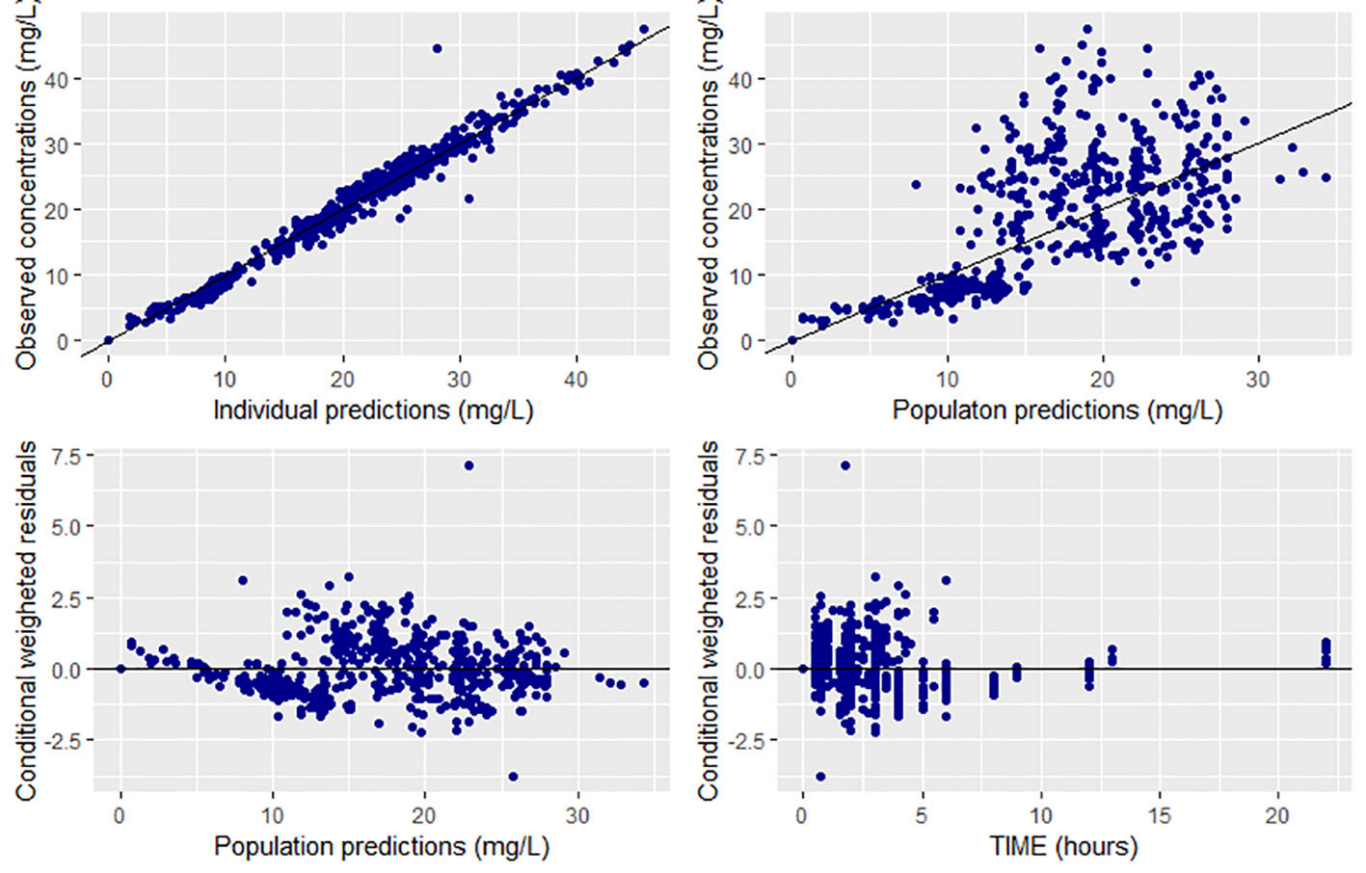
Goodness of fit plots for the final population PK model of CPA (prediction-based (top panel) and residual based (bottom panel) goodness of fit plots.

**Figure 2:**
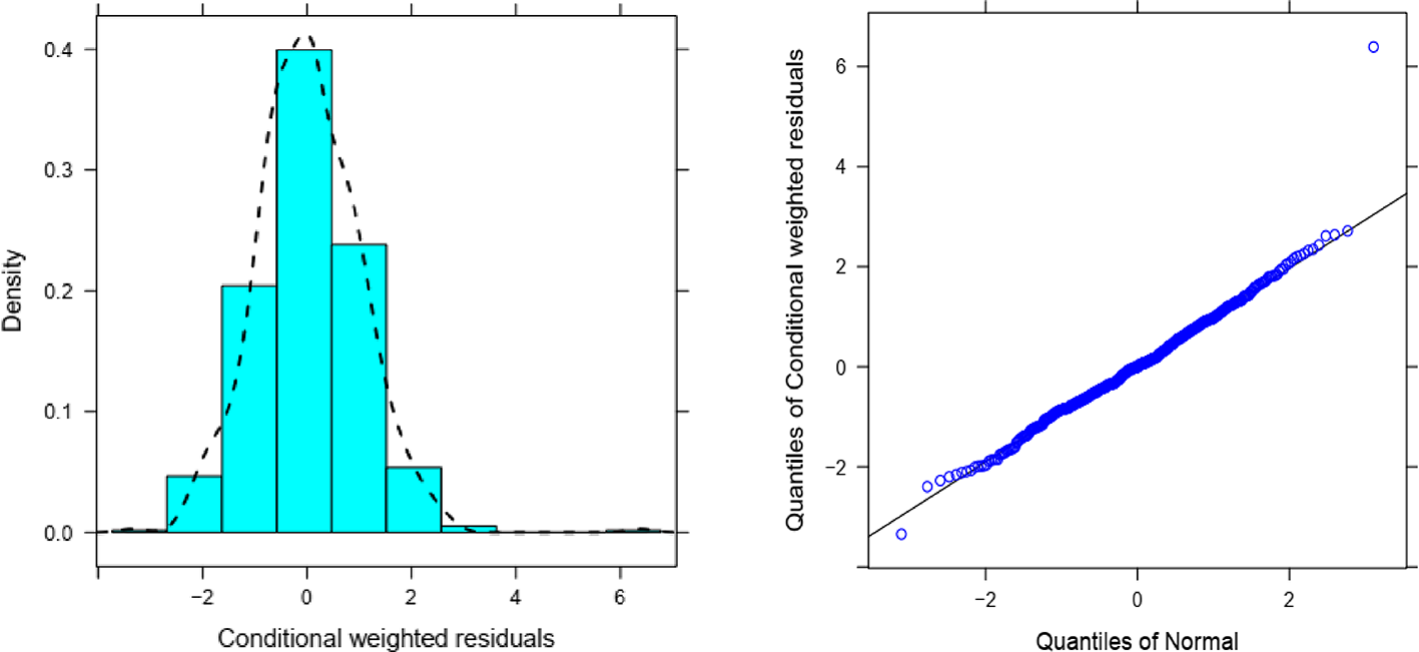
The distribution (histogram (left) and QQ plots (right) of CWRES.

**Figure 3:**
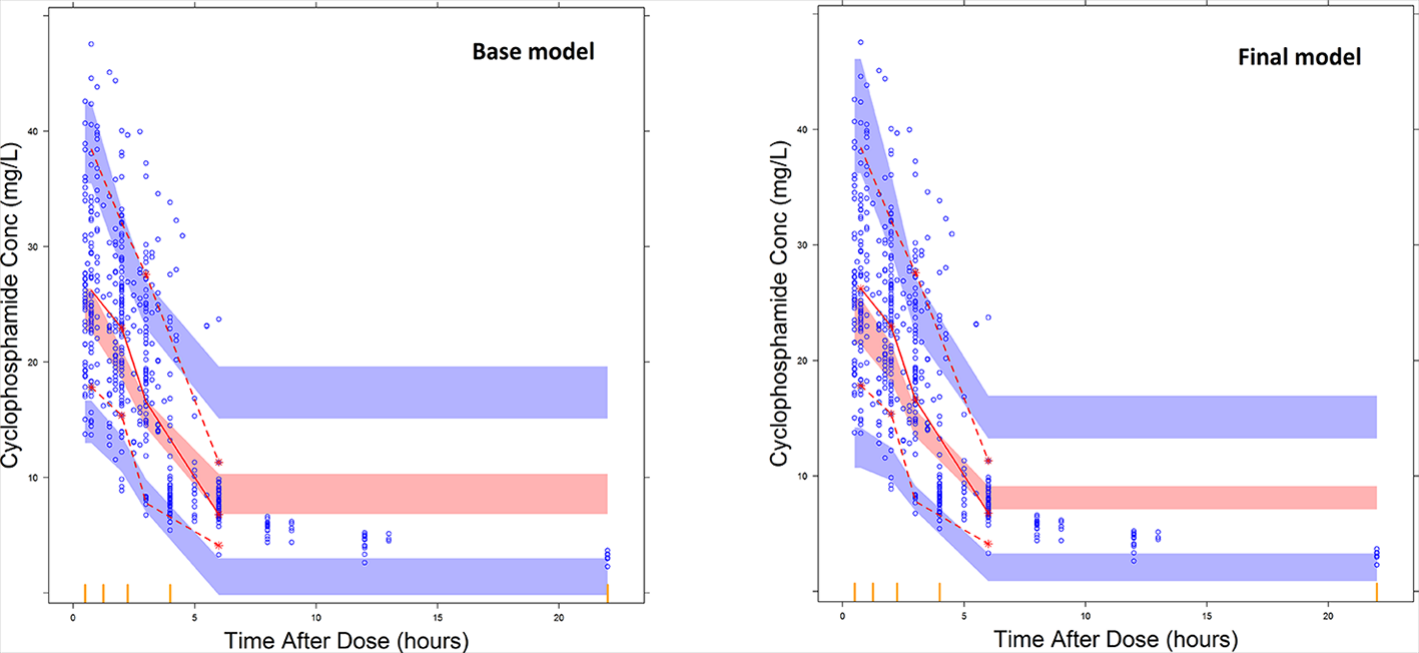
Prediction corrected visual predictive check plots for the base model and final covariate models. The blue dots represent the distribution of observed plasma CPA concentrations and the broken lines represent the 5^th^ (bottom) and 95^th^ percentile (top line) of the median observed data. The shaded regions represent the 95% prediction interval for the observed concentrations.

Overall, goodness of fit plots shows that one compartment model describes the observed plasma concentrations adequately (**Figure 1**). The plot of observed concentration *vs*. population predicted concentration show uniformly distributed points on either side of lines of identity (**Figure 1** top panel). The conditional weighted residuals (CWRES) are within acceptable range and are uniformly spread when CWRES are plotted against population predictions and also against time (**Figure 1**, bottom panel). Moreover, QQ plot and histogram of CWREs indicated normal distribution demonstrating good fit (**Figure 2**). Analysis of prediction corrected visual predictive check (pcVPC plot) showed that, for the base and final model, the observed concentrations are within the 5^th^ and 95^th^ % of the prediction intervals. However, concentrations are under-predicted at lower concentrations (**Figure 3**).

Covariate testing in NONMEM identified CPA dosage regimen (CPA 500 mg/m^2^ or 600 mg/m^2^ based), as significant predictor of clearance and V_D_ of CPA. BSA was also significantly associated with V_D_ of CPA (**Table 4**). Accordingly, 500 mg/m^2^ based CPA regimen was associated with a 32.3% lower than average clearance and 37.1% lower than average V_D_ of 600 mg/m^2^. Similarly, a 0.1 m^2^ unit increase in BSA was associated with a 5.6% increment in V_D_ of CPA.

**Table 4:** Covariate model analysis of cyclophosphamide.

Parameters	Covariate	Covariate model	
		Covariate effect	THETA estimate
Clearance (CL)	CPA^♣^ dose		
	500 mg/m^2^	(1 + THETA1)	-0.323
	600 mg/m^2^	1 (ref)	
Volume (V_D_)	BSA^£^	(BSA/1.58)**THETA2	0.861
	CPA dose		
	500 mg/m^2^	(1 + THETA1)	-0.371
	600 mg/m^2^	1 (ref)	

^£^BSA body surface area; ^♣^ *CPA* cyclophosphamide.

On the other hand, analysis of variance showed statistically significant difference in mean V_D_ between BMI groups (*p* < 0.001). The mean V_D_ (33.5 L) in underweight group (BMI < 18.5 kg/m^2^) was significantly lower compared to overweight group (BMI 25.0–29.9 kg/m^2^) (48.1 L) (*p* < 0.001) or obese patients (BMI ≥ 30 kg/m^2^) (51.9 L) (*p* < 0.001). In patients who received 600 mg/m^2^, but not in 500 mg/m^2^, K_el_ and t_1/2_ of CPA was significantly different between BSA (*p* < 0.001, *ANOVA*). *Post hoc* analysis showed that patients with BSA > 1.75 m^2^ had a longer half-life (6.71 h) compared to those with BSA < 1.5 m^2^ (4.78 h) (*p* < 0.001) or BSA between 1.5 and 1.74 m^2^ (5.42 h) (*p* = 0.004).

**Figure 4:**
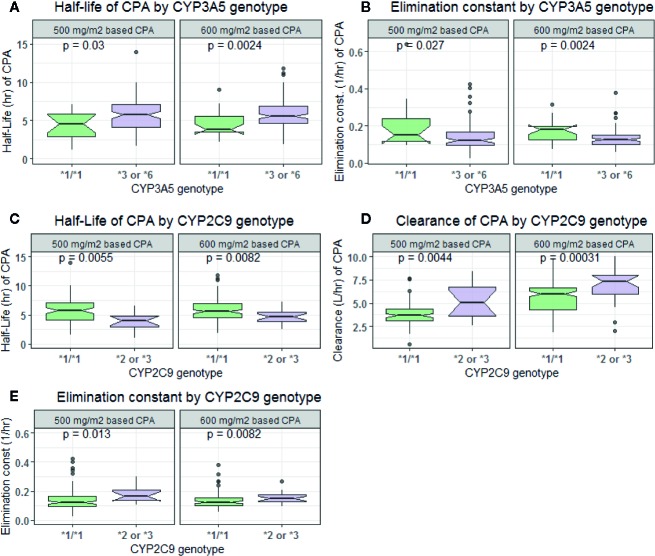
Comparison of clearance, elimination constant and half-life of cyclophosphamide by *CYP3A5* **(A**, **B)** and *CYP2C9* **(C**–**E)** genotypes.

**Figure 5:**
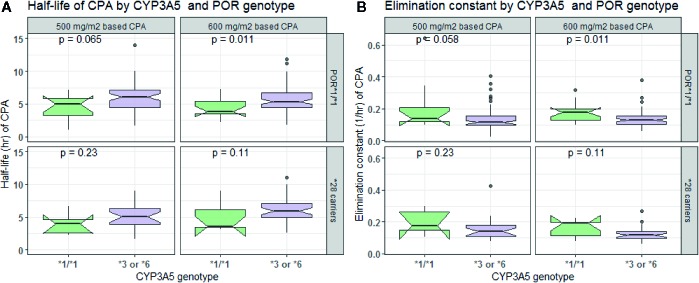
Comparison of half-life **(A)** and elimination constant **(B)** of cyclophosphamide by *CYP3A5* and *POR* genotype.

**Figure 6:**
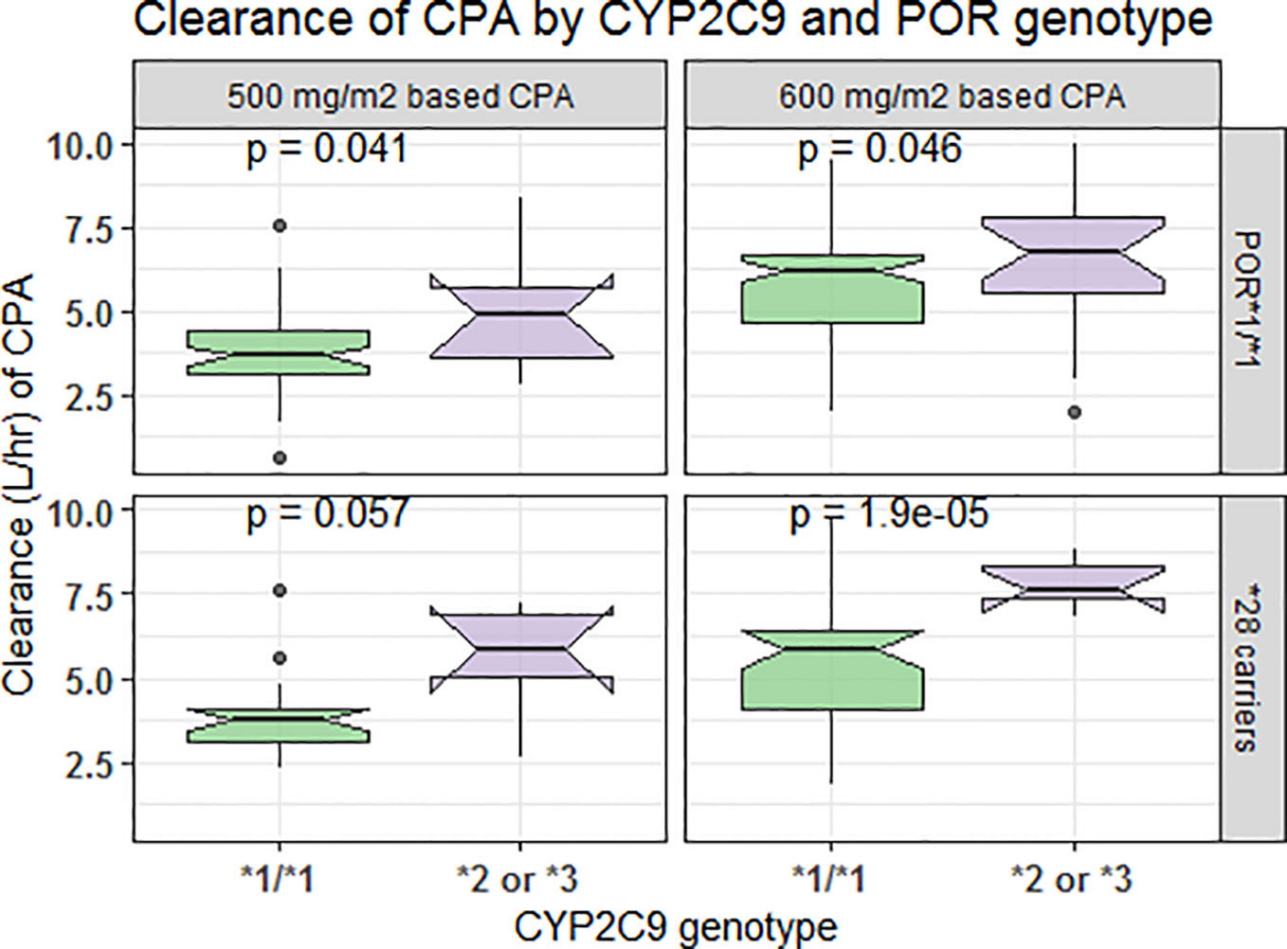
Comparison of clearance of cyclophosphamide by *CYP2C9* and *POR* genotype.

In comparison to those with CYP3A5*1/*1 genotype, patients carrying CYP3A5*3 or *6 alleles had a lower elimination rate constant (0.128 *vs.* 0.179 h^−1^ for 500 mg/m^2^ and 0.124 *vs.* 0.16 h^−1^ for 600 mg/m^2^ group) and longer half-life (5.42 *vs*. 3.88 h for 500 mg/m^2^ and 5.58 *vs*. 4.34 h for 600 mg/m^2^ group) (*p* < 0.001, *t*-test) (**Figures 4A, B**). Further controlling for effect of *POR* genotype, the difference in mean K_el_ or t_1/2_ between *CYP3A5* genotypes was evident among those with POR*1/*1, but not in POR*28 carriers, in both CPA regimen groups (**Figure 5**). CYP2C9 *2 or *3 carriers were also associated with increased clearance of CPA and reduced half-life (**Figures 4C, D**). On the other hand, in both CPA regimen group, with POR*1/*1 genotype, clearance of CPA was moderately associated with *CYP2C9* genotype. In patients carrying POR*28, increased clearance was significantly associated in carriers of CYP2C9 *2 or *3 alleles (**Figure 6**).

### Pharmacodynamic Modeling

**Figure 7:**
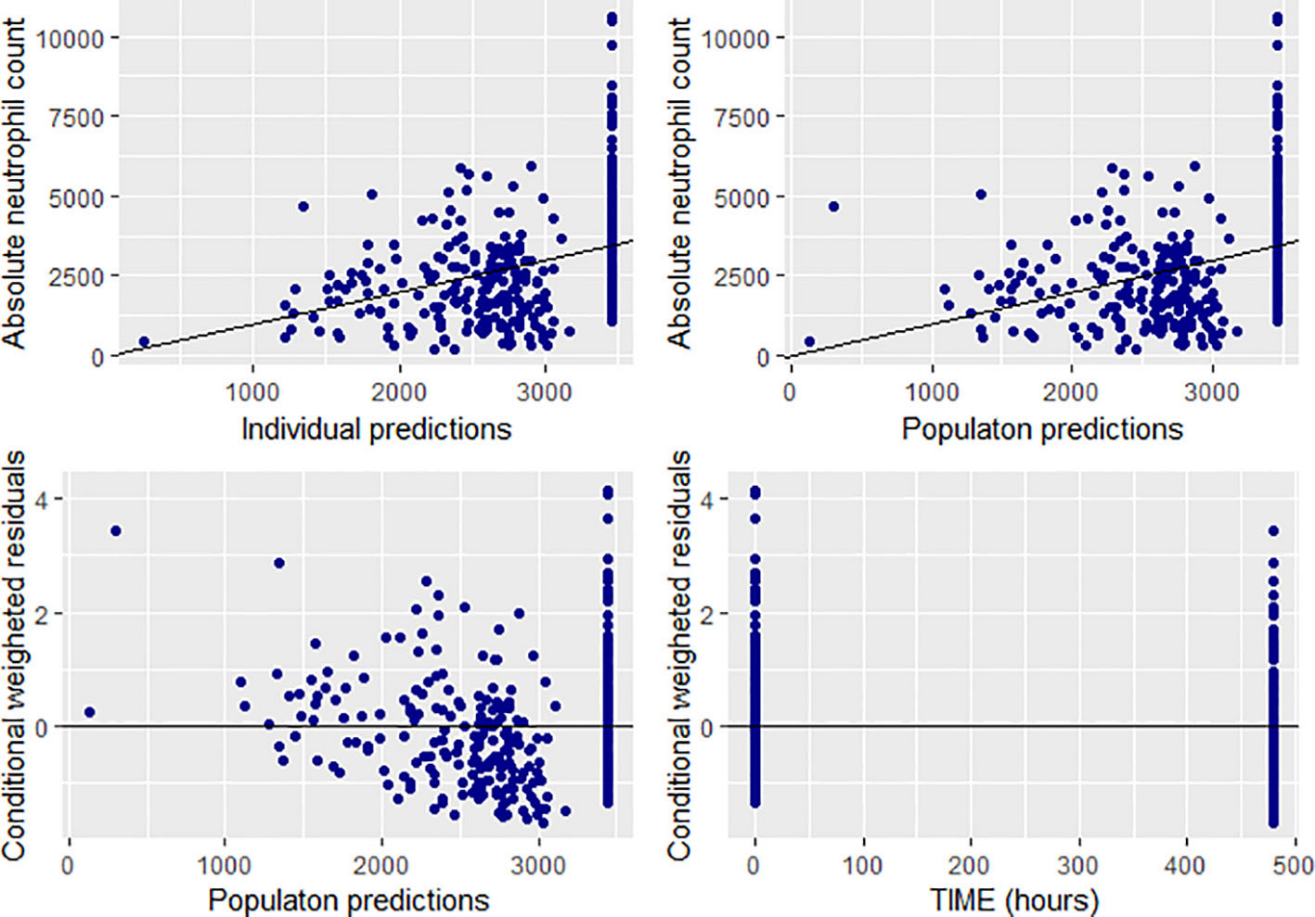
Goodness of fit plots for the final population PD model (absolute neutrophil count, individual and population predictions were all in 10^3^ cells/mm^3^).

The linear direct response (empiric) model was successfully fitted to the neutrophil count. The goodness of fit plots is depicted in **Figure 7**. Combined proportional and additive error model performed well compared to the additive or proportional error models. The parameter estimates for the slope, and ANC_o_ were −1.42 and 3,450, respectively. Accordingly, a one unit increase in the AUC of CPA was associated with a decrease in the neutrophil count by 1.42.

## Discussion

In the present study, we developed population pharmacokinetics, pharmacogenetics, and pharmacodynamics (PK-PG-PD) model for CPA in Ethiopian breast cancer patients at conventional CPA regimen. Our major findings include i) PK of CPA was adequately described by one-compartment model, with estimated population clearance of 5.41 L/h and V_D_ of 46.5 L, ii) presence of large inter-individual variability in CPA clearance (54.5%), iii) CPA dosage regimen, BSA, BMI, *CYP2C9*, and *CYP3A5* genotypes significantly influence PK of CPA, and iv) a positive correlation between CPA exposure (AUC) and neutropenic toxicity. To our knowledge, this is the first study to investigate the population PK-PD model for CPA in breast cancer patients from sub-Saharan Africa.

Our result indicates that one-compartment model better described CPA concentration data, which is in agreement with previous reports (Timm et al., 2005; Joerger et al., 2007). In contrast, other studies described PK parameters using two-compartment model (Nakajima et al., 2007; Ekhart et al., 2008). These findings may suggest that the simple one or two-compartment models may be sufficient to describe PK parameters in conventional doses of CPA administered by short IV infusion. The differences in the models (one *vs.* two compartmental models) could be related to phenomenological arguments (for example the physico-chemical properties of CPA) as well as mathematical arguments (Sandulovici et al., 2009).

On the other hand, earlier studies demonstrated that when CPA is administered in a high dose regimen (*e.g*., 4,000 mg/m^2^) and by short infusion, a concentration-dependent PK model of CPA was developed with convex downward elimination curves (Chen et al., 1995; Chen et al., 1997). A time-dependent PK model was also described in patients receiving high dose CPA (6,000 mg/m^2^) in a prolonged infusion (96 h) (Chen et al., 1995). Moreover, other studies developed a PK model for CPA incorporating the effect of auto-induction (Hassan et al., 1999) and the phenomena of both auto-induction and drug-drug interaction between CPA and thioTEPA (Huitema et al., 2001). These findings show that, in addition to other factors, PK of CPA could be influenced by the dosing history and the duration of IV infusion.

The volume of distribution estimated in this study (46.5 L) is also in agreement with a previous report (de Jonge et al., 2005). Given that one-compartment PK model described our data, together with V_D_ which approximates to the total body water; it is plausible that CPA achieves instantaneous distribution throughout the body. The population clearance of 5.41 L/h estimated in the present study is also in agreement with those reported in breast cancer patients from France (Joerger et al., 2007) but higher compared to the finding from Swedish and Japanese breast cancer patients (Hassan et al., 1999; Nakajima et al., 2007; Ekhart et al., 2008). Indeed, higher CYP enzyme activities including *CYP3A* in Ethiopian population compared to others have been reported previously (Gebeyehu et al., 2011; Aklillu et al., 2014), which may explain the higher CPA clearance rate in Ethiopians than Swedish or Japanese breast cancer patients.

After covariate analysis in NONMEM, CPA regimen caused a reduction of coefficient of variation from 54.5 to 46.4% and thus accounting for 8.1% of the variability. Thus, larger part (46.4%) of the variability in CPA clearance remains unexplained. Similarly, substantial variations in PK and exposure to CPA and its metabolites have been reported previously in both adults and children (de Jonge et al., 2005). These variations could be explained either by the differences in the level of expression of specific CYP enzymes or the level of activity of the individual enzymes (Zanger and Schwab, 2013) or other non-genetic factors (de Jonge et al., 2005). Results of previous studies regarding the roles of the individual CYP enzymes’ genes to PK differences of CPA are inconsistent. In this study, the variant alleles in *CYP2B6*, *CYP2C19*, *CYP2J2*, and *ABCB1* were not associated with PK of CPA. In agreement with our finding, previous studies reported that polymorphisms in *CYP2B6* and *CYP2C19* did not have significant role in variations of CPA PK (Ekhart et al., 2008; Fernandes et al., 2011; Afsar et al., 2012; Raccor et al., 2012). The lack of association of *ABCB1* genotype was reported by a previous study (Kim et al., 2013). The inconsistent findings from the literature regarding the impact of pharmacogenetic variation on the PK parameters of CPA could be due to differences in the study population, environmental factors, extent of enzyme induction, or involvement of multiple competing CYP pathways involved in the biotransformation and clearance of CPA such as *CYP2J2*, *CYP2C9*, *CYP2C19*, *CYP3A4*/*5*, *CYP2A6* (Pinto et al., 2009). Between population variation in CYP enzyme activity and importance of environmental factors is well recognized (Aklillu et al., 2002; Hatta et al., 2015). For instance, unique distribution variant alleles and higher CYP enzyme activities particularly in Ethiopians compared to other population is reported for *CYP2C9* (Scordo et al., 2002), *CYP2C19* (Sim et al., 2006), *CYP2B6* (Ngaimisi et al., 2013), *CYP2A6* (Aklillu et al, 2014), and *CYP3A* enzymes (Gebeyehu et al., 2011). Indeed, having the same genotype, higher *CYP2B6* enzyme activity in Ethiopians than Tanzanians is reported (Ngaimisi et al., 2013). Black Africans are the most genetically divers population on earth and hence extrapolation pharmacogenetic findings from white or Asian population to black African population may not be applicable always.

On the other hand, the findings of Xie et al. demonstrated, using human liver microsomes, that patients with *CYP2B6* G516T variants had a two-fold higher CPA clearance compared to those carrying the wild type (Xie et al., 2003). In Japanese breast cancer patients, those homozygous for CYP2B6*6 (Q172H and K262R) had higher clearance than heterozygous or homozygous for CYP2B6*1 (Nakajima et al., 2007). In another study, CYP2C19*2 variant allele was associated with lower elimination rate constant in individuals receiving < 1,000 mg/m^2^ of CPA (Timm et al., 2005), which was not evident at higher doses. Notably, a significantly increased elimination rate of CPA was observed at CPA doses > 1,000 mg/m^2^, possibly due to CYP induction at higher doses (Timm et al., 2005). In our study, a dose-dependent clearance of CPA was also observed. Patients receiving 600 mg/m^2^ had significantly higher clearance as compared to 500 mg/m^2^ CPA dosing (5.81 *vs*. 3.97 L/h, *p* < 0.001). CPA induces *CYP2C* and *CYP3A4* in human hepatocytes by increasing the enzyme production rate and thereby increasing the amount of enzyme by which CPA is metabolized (Hassan et al., 1999).

The present study also revealed that carriers of CYP3A5*3 or *6 had lower mean elimination rate constant and prolonged half-life compared to those with CYP3A5*1/*1 genotype (*p* < 0.001). In contrast, CYP2C9 *2 or *3 carriers were associated with increased clearance of CPA. The observed effect of *CYP2C9* on CPA clearance was unexpected, as CYP2C9 *2 or *3 alleles are defective variant alleles associated with reduced *CYP2C9* enzyme activity resulting in reduced clearance of the drug. However, our finding is supported by Balasubramanian et al. that reported that CYP2C9 *2 allele increased CPA clearance and decreased its AUC (Balasubramanian et al., 2009). These findings may suggest that the extent and pattern of CYP activity and induction or inhibition may be variable among various patient populations. For example, POR*28 was demonstrated to significantly alter *CYP2C9* activity in Swedish, but not in Korean healthy subjects (Hatta and Aklillu, 2015). In the present study, increased CPA clearance was significantly higher in carriers of CYP2C9 *2 or *3 alleles who also carry POR *28 allele. In both CPA regimen group and with POR*1/*1 genotype, clearance of CPA was moderately associated with *CYP2C9* genotype. Moreover, multiple competing CYP pathways involved in the biotransformation and clearance of CPA might also play a prominent role in *CYP2C9* defective patients, compensating for the absence of *CYP2C9* activity (Gaedigk et al., 2018). The relative contribution of pathways may shift in the presence of an inducer. CYP3A4 for instance may be a minor pathway under normal circumstances, but may become more prominent when a *CYP3A4*-inducing agent is co-administered (de Leon et al., 2008). Furthermore, the reported higher CYP enzyme activities including *CYP3A* (Gebeyehu et al., 2011; Aklillu et al., 2014), *CYP2B6* (Ngaimisi et al., 2013), *CYP2C9* (Scordo et al., 2002), *CYP2C19* (Sim et al., 2006), and *CYP2A6* (Aklillu et al, 2014) in Ethiopian population compared to others may explain the higher CPA clearance rate in the present study population. Indeed, having the same genotype, higher *CYP2B6* enzyme activity in Ethiopians than Tanzanian is also reported previously (Ngaimisi et al., 2013).

A previous study demonstrated that *CYP2J2* expression was significantly up-regulated during CPA treatment and the bio-activation of the drug was significantly correlated to *CYP2J2* expression (El-Serafi et al., 2015a). *CYP2J2*, expressed in high levels particularly in extra-hepatic organs such as the heart, intestine, and urinary bladder, may be responsible for the local CPA bio-activation and may explain CPA treatment-related toxicities (Hashizume et al., 2002; Matsumoto et al., 2002; Lee et al., 2010). In a recent cohort study investigating the association of pharmacogenetic variations to hematologic toxicity in Ethiopian breast cancer patients, we reported that patients carrying CYP2J2*7 variant alleles were associated with higher incidence of hematological toxicity (Ahmed et al., 2019). However, the present study could not identify association of *CYP2J2* genetic polymorphism and PK of first cycle CPA. The discrepancy could be explained by the increased *CYP2J2* expression as well as CYP induction in the subsequent chemotherapy cycles, which could facilitate formation of active metabolite and contributing for the increased incidence of hematologic toxicity observed in the course of the chemotherapy cycles.

Cytochrome P450 oxidoreductase (*POR*), a flavoprotein that supplies electrons to all CYP enzymes for their catalytic activity (Hart et al., 2008), was implicated to influence CYP catalyzed bio-activation of xenobiotics (Hu et al., 2012). In this study, *POR* was found to alter *CYP3A5* activity. Among patients with POR*1/*1, the mean elimination constant (K_el_) or half-life (t_1/2_) between *CYP3A5* genotypes was significantly different. However, the effect was not evident in POR*28 carriers. A previous study demonstrated a significant correlation between CPA clearance and *POR/CYP* ratio (El-Serafi et al., 2015b), which might signify the importance of the level of expression of the gene in CPA metabolism and clearance.

The major toxic effect of CPA at conventional doses and the primary reason for a change in the schedule of CPA based chemotherapy delivery to breast cancer patients are treatment associated neutropenia (Kozma et al., 2012). Consequently, one aspect of optimizing cancer chemotherapy involves describing neutropenic toxicity as a function of drug exposure. The present study demonstrated a positive relationship between AUC of CPA and neutropenic toxicity. A previous study also showed that breast cancer patients with complete response had significantly higher CPA AUC than patients with progressive disease (Joerger et al., 2007). The greater part of total systemic clearance of CPA is non-renal clearance (de Jonge et al., 2005), and hence, these findings may suggest that exposure to CPA may be predictive of the amount of activated metabolite formed. High exposure to bioactivated CPA was also reported to relate to the occurrence of venous-occlusive disease of the liver following high-dose CPA (de Jonge et al., 2006). In contrast, an inverse association was also reported between the AUC of CPA and its treatment-related cardiotoxicity and event-free survival in women with breast cancer (Ayash et al., 1992; Petros et al., 2002). Patient with significantly lower AUC developed congestive heart failure in a high-dose CPA regimen (Ayash et al., 1992). Treatment effectiveness and occurrence of adverse effects like heart failure were also inversely related to CPA AUC in high-dose therapy (Petros et al., 2002). On the other hand, other studies could not establish association between CPA exposure and toxicity or relapse free survival in cancer patients treated with high-dose chemotherapy (Nieto et al., 1999; Veal et al., 2016). The discrepancies in the results of the various studies could be attributed to the differences in the sample size, patient population, and richness of the PK and PD data in the individual studies.

The present study describes the population PK and PD of the first cycle CPA in a resource-limited setting. However, important limitations are also to be noted. First, the metabolite concentration was not determined and consequently, it was not possible to incorporate it in the PK-PD model. In addition, as CPA is commonly used in combination with other chemotherapeutic agents in breast cancer treatment, their contribution in the observed neutropenic toxicity would be expected. Moreover, concentration and response were collected at different time points. Owing to time-dependent signal transduction in hematopoiesis, the simple empiric model adopted in this study may not account for this time lag between drug concentration and observed effects of CPA and the results also may not reflect the underlying physiological process. The PD observations (data points) in our study were also few (taken at baseline and day 20 post-CPA administration) which might have hampered the successful development of the semi-mechanistic model.

In conclusion, the PK profile of CPA is described by one-compartment model in Ethiopian breast cancer patients. CPA displays wide between patient variability in clearance, which is partly explained by variation in CPA dosage regimen, BSA, BMI, *CYP3A5,* and *CYP2C9* genotypes. CPA plasma exposure predicts chemotherapy-associated neutropenic toxicity. Further studies are recommended to explore the PK of the metabolite (4-hydroxycyclophosphamide), incorporating auto-induction by CPA and drug interaction and correlate its impact in neutropenic toxicity. Moreover, PK-PD modeling involving the contribution of other anticancer drugs in CPA containing regimen needs to be explored.

## Data Availability Statement

The raw genotyping and patient clinical data underlying the conclusions of this article are not publicly available as permission to do so was not included in the protocol approval granted by the ethics committee. The raw data supporting the conclusions of this article will be made available by the authors, without undue reservation, to any qualified researcher.

## Ethics Statement

The studies involving human participants were reviewed and approved by Institutional Review Board of the College of Health Sciences, Addis Ababa University, Armauer Hansen Research Institute Ethical Review Committee National Research Ethics Review Committee of the Federal Democratic Republic of Ethiopia. The patients/participants provided their written informed consent to participate in this study.

## Author Contributions

JA, EM, and EA designed the study. JA collected the data. JA and EA did the genotyping. JA, RB, and EA analyzed the data and drafted the manuscript. JA, EA, EM, RB, JM, AA, RH, AF, and MH involved in the discussion of results and critical review of the manuscript. All the authors have read and approved the final manuscript.

## Funding

This research was supported by the Armauer Hansen Research Institute (AHRI) through the BSPP Program, a grant obtained from Sida-Ethiopia Bilateral Program (Contribution No: 5108013506). The funders had no role in the design of the study; in the collection, analyses, or interpretation of data; in the writing of the manuscript, or in the decision to publish the results.

## Conflict of Interest

The authors declare that the research was conducted in the absence of any commercial or financial relationships that could be construed as a potential conflict of interest.

## Abbreviations

ALP, alkaline phosphatase; ALT, alanine aminotransferase; ANC, absolute neutrophil count; ANOVA, analysis of variance; AST, aspartate aminotransferase; AUC, area under the curve; BMI, body mass index; BSA, body-surface area; BUN, blood urea nitrogen; CI, confidence intervals; CL, clearance; CPA, cyclophosphamide; CV, coefficient of variation; CWRES, conditional weighted residuals; FOCE, first-order conditional estimation; GOF, goodness of fit; HWE, Hardy-Weinberg equilibrium; HIV, human immunodeficiency virus; IIV, inter-individual variability; IRB, Institutional Review Board; K_el_, elimination constant; NONMEM, non-linear mixed effect modeling; OFV, objective function value; pcVPC, prediction corrected visual predictive check; PD, pharmacodynamics; PG, pharmacogenetics; PK, pharmacokinetics; SCM, stepwise covariate modeling; SCr, serum creatinine; t_1/2_, half-life; V_D_, volume of distribution; WBC, white blood cells.
